# One giant leap for mankind: The experience of studying medicine through the pandemic

**DOI:** 10.15694/mep.2020.000274.1

**Published:** 2020-12-08

**Authors:** Astrid Nieto, Mildred López

**Affiliations:** 1 Tecnologico de Monterrey

**Keywords:** medical students, medical education, pandemic, COVID-19, coronavirus, anxiety, educational experience, learning environment, undergraduate

## Abstract

This article was migrated. The article was marked as recommended.

When the COVID-19 pandemic started, a well-intentioned person told me that studying medicine in times of coronavirus was like being an astronaut when humanity first landed on the moon. Space meant for astronauts the clash of their passion for discovery and learning, with the risk of not coming alive back home to their loved ones. Health professionals have experienced the same this year in the front line against coronavirus. After eight months of this new normality, more than one million lives have been lost worldwide, and they carry on their shoulders the hope of millions of people optimistic about the outcome of their effort.

Medical students, even at an early stage of their training, are aware of the damage this virus causes, but their lack of clinical experience has limited them to be involved in direct patient care. They have not been able to honor the call of a vocation of service to the community, and it has made them experience the shame of leaving on their own to other health professionals in this historic challenge.

## Introduction

When the COVID-19 pandemic just started, a well-intentioned person told me that studying medicine in times of coronavirus was like being an astronaut when humanity first landed on the moon. However, I have serious doubts about commander Armstrong felt as many medical students do today. Multiple articles published amid the pandemic highlight the challenging experiences in the clinical environment ( Sánchez-Duque, 2020), the demands from transitions to remote emergency teaching ( Núñez-Cortés, *et al.*, 2020), and the social responsibilities of medical schools in the face of this emergency ( Abreu-Hernández *et al.*, 2020). Nevertheless, the perspective of medical students in the early years of the program has rarely been discussed.

Similarly to the space race, the pandemic has been loaded with scientific advances and technological developments which has contributed to the improvement of digital platforms, the formalization of telemedicine, and the incorporation of new learning and assessment strategies for education. These transformations happened in record time and were key factors for students to continue their educational programs and become the next generation of physicians.

I can imagine that as the Apollo-11 astronauts were preparing for their mission, the crew did feel excited about their journey ahead, but worried because they knew that there would be challenges in entering one of the most extreme environments imaginable. Space meant the clash of their passion for discovery and learning, and the risk of not coming alive back home to their loved ones. It is very similar to what health professionals have experienced this year in the front line against coronavirus, they carry on their shoulders the hope of millions of people optimistic about the outcome of their effort.

After eight months of this new normality, the toll on society has been enormous, more than one million lives have been lost worldwide, and more than 45 million cases have been reported. Medical students, even at an early stage of their training, are aware of the damage that this virus causes to the body, nevertheless, their lack of clinical experience prevents them to be involved in direct patient care. By not being able to take this *one small step for man*, their experience is closer to what the Apollo-13 crew went through. They are witnesses of an extreme situation in which they can not take part which makes them experience the frustration of not being able to *honor the call* of a vocation of service to the community and also shame for feeling that they are leaving on their own to the future colleagues in health professions in this historic challenge.

## Take Home Messages

• COVID-19 transition to a remote educational model has been challenging for the whole educational community: students, educators, administrators, and their families.
• Pandemic adaptation of the learning environment must reach the social dynamics of education, rather than focusing exclusively on content delivery.
• More studies should address the wide-impact of the health crisis of early-stage students in the undergraduate program.

## Notes On Contributors

**Astrid Nieto** is a third-year medical student. She is a member of Mentor Students of Excellence (MAES) group where she gives peer tutoring and classes, where she became intrigued by medical education. She is passionate about medicine, and how the improvement of technologies can help reduce carbon footprint in the healthcare sector. She is also part of the American Association of Neurosurgeons (AANS) and dreams to become one.

**Mildred López** is a Professor and Director of Educational Innovation at the School of Medicine and Health Sciences of the Tecnológico de Monterrey. Ph.D. in Educational Innovation and MSc in Quality and Productivity. Dr. López is a Medical Education Fellow of the Foundation for the Advancement of International Medical Education and Research (FAIMER) Institute, and the Association for Medical Education Europe (AMEE). An active member of the Latin American Federation for Clinical Simulation and Patient Safety (FLASIC) and founding member of the Healthy Living for Pandemic Event Protection (HL - PIVOT) network.
