# Supplementary material for: One giant leap for mankind: The experience of studying medicine through the pandemic
Source: MedEdPublish (2016). 2020 Dec 8;9:274. [Version 1] doi: 10.15694/mep.2020.000274.1 (PMC10697489; doi:10.15694/mep.2020.000274.1)
Supplement: Peer review history

# Reviewer report: Reviewer response for version 1

**DOI:** 10.21956/mep.20206.r31281

**Published:** 2021-03-06

**Authors:** Ken Masters

**Affiliations:** 1 Sultan Qaboos University

This review has been migrated. The reviewer awarded 3 stars out of 5 An opinion piece on the experience of studying medicine through the pandemic.While it does begin well, and has the kernel of a good paper, I think the piece would have been strengthened by more reflection on the students’ feelings to give deeper insight beyond only the surface ideas. I think that the authors are not entirely correct when they write “Nevertheless, the perspective of medical students in the early years of the program has rarely been discussed.” These perspectives have been rather widely discussed. It may be that the authors are using the term “the early years of the program” in a specific sense or are referring to specific instances; in which case, it would be useful to expand upon this, and give more details.So, the piece is the start of a valuable insight, but could benefit from some further development.

Reviewer Expertise:

NA

No decision status is available

# Reviewer report: Reviewer response for version 1

**DOI:** 10.21956/mep.20206.r31283

**Published:** 2020-12-17

**Authors:** Camilo Torres

This review has been migrated. The reviewer awarded 4 stars out of 5 The article is a good analogy of the reality that medical students in the early years have been exposed during these months of pandemic and adaptation to this new reality. Some ideas are raised concerning the emotions they have had to face, especially the frustration of not being able to be contributing more actively.The text is clearly written and is enjoyable to read. My suggestion is that it would have had a greater development of the feelings that the students have presented during this time and have concluded with some reflections or recommendations.

Reviewer Expertise:

NA

No decision status is available

# Reviewer report: Reviewer response for version 1

**DOI:** 10.21956/mep.20206.r31282

**Published:** 2020-12-12

**Authors:** Mary Higgins

**Affiliations:** 1 University College Dublin, National Maternity Hospital

This review has been migrated. The reviewer awarded 2 stars out of 5 This is a lovely reflection and thank you both for writing it. You may be interested in reading some of the reflections that the BMJ published by medical students about their experiences and perceptions of the conflicts of being a student during the pandemic, that really illustrate the themes that you have outlined. A critical reflection in the context of these themes would be very meaningful. I also sincerely believe that we should all reflect on how we can maintain patient focused education through virtual learning, as the patient should always be the focus of our teaching, our learning and our care.

Reviewer Expertise:

NA

No decision status is available
